# Two-photon excited luminescence of structural light enhancement in subwavelength SiO_2_ coating europium ion-doped paramagnetic gadolinium oxide nanoparticle and application for magnetic resonance imaging

**DOI:** 10.1186/s11671-023-03864-y

**Published:** 2023-06-13

**Authors:** Wei Wang, Shangling Song, Wendong Liu, Tong Xia, Gang Du, Xiangyu Zhai, Bin Jin

**Affiliations:** 1 Medical Integration and Practice Center, Shandong University, Jinan, Shandong China; 2 Medical Equipment Department, The Second Hospital of Shandong University, Jinan, Shandong China; 3 Department of Hepatobiliary Surgery, The Second Hospital of Shandong University, Jinan, Shandong China; 4 Organ Transplant Department, Qilu Hospital of Shandong University, Jinan, Shandong China

**Keywords:** Two-photon absorption, Optical nonlinear, Rare-earth ion luminescence, Dual-modal bioimaging, MRI contrast agent

## Abstract

**Background:**

Oxides of lanthanide rare-earth elements show great potential in the fields of imaging and therapeutics due to their unique electrical, optical and magnetic properties. Oxides of lanthanide-based nanoparticles enable high-resolution imaging of biological tissues by magnetic resonance imaging (MRI), computed tomography (CT) imaging, and fluorescence imaging. In addition, they can be used to detect, treat, and regulate diseases by fine-tuning their structure and function. It remains challenging to achieve safer, efficient, and more sensitive nanoparticles for clinical applications through the structural design of functional and nanostructured rare-earth materials.

**Result:**

In this study, we designed a mesoporous silica-coated core–shell structure of europium oxide ions to obtain near-infrared two-photon excitation fluorescence while maintaining high contrast and resolution in MRI. We designed enhanced 800 nm photoexcitation nanostructures, which were simulated by the finite-difference method (FDM) and finite-difference time-domain method (FDTD). The nanoparticle structure, two-photon absorption, up-conversion fluorescence, magnetic properties, cytotoxicity, and MRI were investigated in vivo and in vitro. The nanoparticle has an extremely strong optical fluorescence response and multiple excitation peaks in the visible light band under the 405 nm continuous-wave laser excitation. The nanoparticle was found to possess typical optical nonlinearity induced by two-photon absorption by ultrafast laser Z-scan technique. Two-photon excited fluorescence of visible red light at wavelengths of 615 nm and 701 nm, respectively, under excitation of the more biocompatible near-infrared (pulsed laser at 800 nm). In an in vitro MRI study, a T1 relaxation rate of 6.24 mM^−1^ s^−1^ was observed. MRI in vivo showed that the nanoparticles could significantly enhance the signal intensity in liver tissue.

**Conclusions:**

These results suggest that this sample has applied potential in visible light fluorescence imaging and MRI.

## Introduction

Nanoparticles are at the forefront of rapidly developing nanotechnology research and have received increasing attention in recent years in the fields of hybrid imaging methods, including fluorescence imaging, ultrasound imaging, computed tomography (CT), positron emission tomography (PET), single photon emission computed tomography (SPECT), magnetic resonance imaging (MRI), etc. [1–5]. Due to their working mechanism, hybrid imaging techniques can exploit the advantages of a single imaging technique while compensating for the disadvantages of each other [6, 7]. Among these techniques, MRI has the advantages of not being limited by the depth of tissue penetration, free of ionizing radiation, high spatial resolution, and capable of performing three-dimensional tomography, but it also has certain limitations in distinguishing certain tissues, especially tiny blood vessels [8, 9]. Several organic and inorganic substances are widely used as contrast agents in clinical MRI scans to improve imaging contrast between normal and diseased tissues [10, 11]. There are two mechanisms to achieve improved imaging, changing the longitudinal relaxation time or the transverse relaxation time, becoming T1 and T2 agents, respectively [12–15]. Improving two or more contrast imaging while reducing toxicity to humans is the driving force behind the development of new functional nanoparticles [16–20].

In the field of biochemical analytical labeling and sensing, the design of specific nanoparticle structures can be used as new diagnostic and therapeutic methods in the medical field [21–23]. Nanoparticles with specific physicochemical properties, quantum size effects and specific surface effects are the main technologies used for fluorescent imaging probes[24]. Among the elements used to synthesize nanoparticles, lanthanides have a relatively long luminescence lifetime and a special 4f–4f transition potential that allows their emission spectra to exhibit narrow and sharp bands (half-maximum width usually less than 10 nm), which are often used to construct fluorescent applications [25, 26]. Nanoparticles synthesized from the lanthanide gadolinium element are currently one of the most effective T1 magnetic resonance contrast agents [27, 28]. Gd^3+^ is divided into 7 unpaired electrons on the 4f electron orbital, which is extremely magnetic. Contrast agents synthesized with gadolinium element have good contrast agent performance in the human body, including chelates of gadolinium (Gd^3+^), oxide nanoparticles, etc. [29–34]. At present, there are some methods to avoid the cytotoxicity of the gadolinium element, such as PEG modification and silica coating. [35–37].

For fluorescence imaging in the hybrid imaging technology, while taking into account the effects of other imaging technologies, it is usually a challenge to control the optical properties of the contrast agent [33]. Lanthanide ions exactly satisfy the requirement of multiple metastable energy levels for the up-conversion luminescence process. Er^3+^, Tm^3+^ and Ho^3+^ with special stepped energy level distribution in lanthanide ions are the most commonly used activating ions [38]. At present, some reports have confirmed that rare-earth ion-doped nanoparticles have excellent up-conversion performance and improve biocompatibility by coating silica, including NaYF_4_, NaGdF_4_, etc., doped rare-earth elements including Yb^3+^, Er^3+^, Tm^3+^, etc., with sizes ranging from tens to hundreds of nanometers [39–42]. Compared to mature carbon-based biomaterials, rare-earth ion-doped nanoparticles are currently the most promising up-conversion two-photon fluorescent materials [43].

**Fig. 1:**
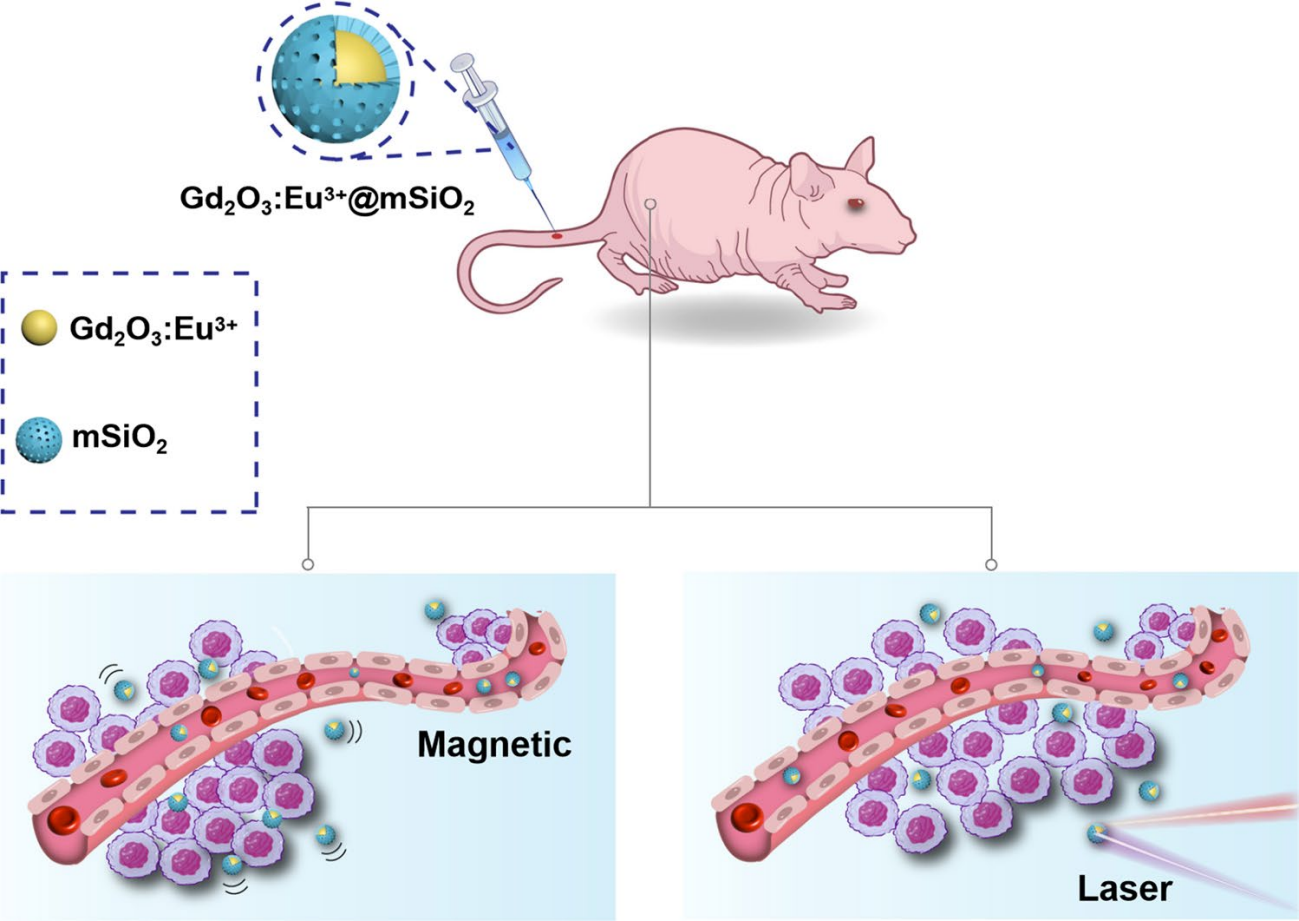
Schematic diagrams of Gd_2_O_3_:Eu^3+^ nanoparticles and dual functions for magnetic and fluorescence applications

In this work, we propose to give a modal of nanostructural design for light field localized to further inducing optical nonlinear effects. A kind nanocomposite of the mesoporous silica-coated paramagnetic gadolinium trioxide was fabricated and a rare-earth luminescent material of europium ion was doped (Fig. 1). Nanoparticles have subwavelength dimensions in the working band of near-infrared-I (NIR-I), in order to achieve visible light fluorescence by a nonlinear process. Mesoporous silica can protect the gadolinium element and can also serve as a release platform for drugs. The nanoparticle samples were fully characterized, including scanning electron microscope (SEM) and photoluminescence (PL) spectroscopy. Under the excitation of a near-infrared 800 nm femtosecond pulsed laser, it showed nonlinear absorption detected by a Z-scan system. As well, it exhibits relatively stable up-conversion fluorescence of visible red light at 615 nm and 701 nm. An in vitro MRI study observed a T1 relaxation rate of 6.24 mM^−1^ s^−1^. Furthermore, in vivo MRI showed that the nanoparticles could significantly enhance the signal intensity in liver tissue. These results suggest that this sample is a promising near-infrared to visible light up-conversion fluorescence imaging and MRI contrast agent with great potential in the diagnosis of various imaging techniques.

## Material and methods

### Optical structure simulation and design

**Fig. 2:**
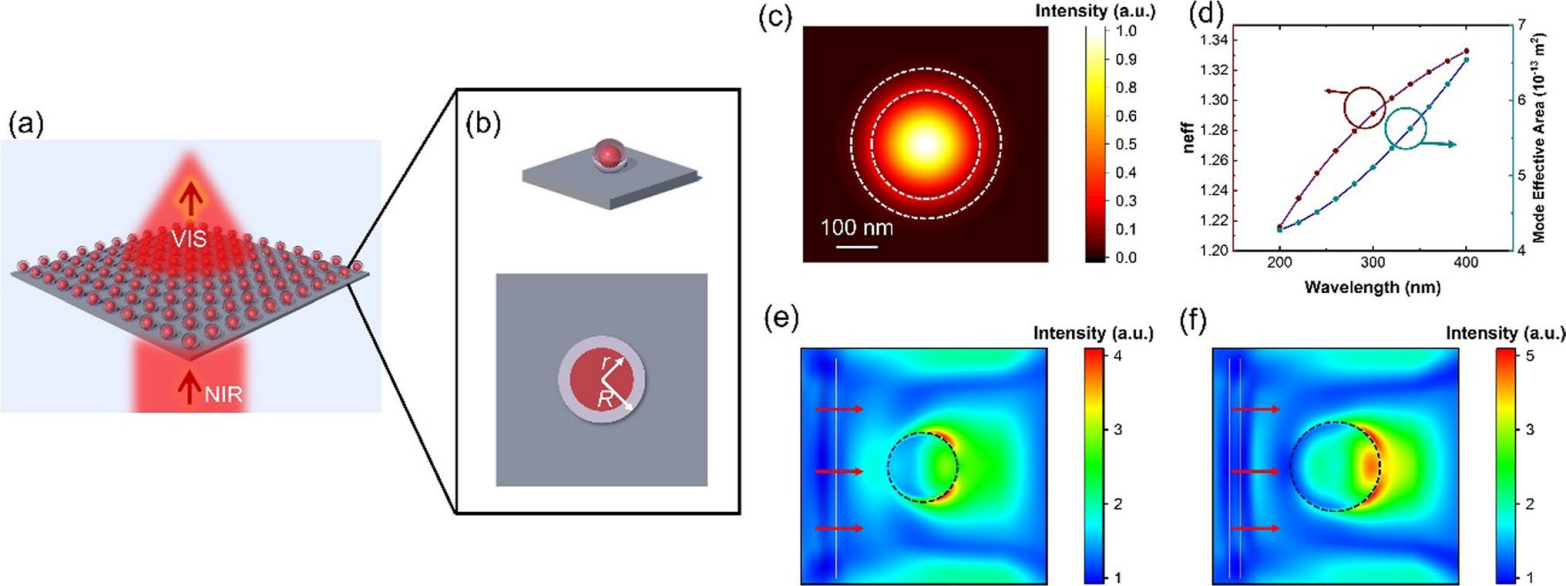
**a** Schematic diagram of the proposed efficiency two-photon excited luminescent nanoparticles capable of emitting visible light luminescent under near-infrared light excitation. **b** Schematic and cross-sectional structure of a single nanoparticle sphere. **c** Optical localized mode field with *R* = 400 nm and *r* = 300 nm. The two white dashed lines mark the SiO_2_ and Gd_2_O_3_ layers of the nanoparticle, respectively. **d** The values of the effective refractive index and the effective area of the mode field in the range of *R* values from 200 to 400 nm. FDTD simulated results of light field distributions with the *R* values of **e** 300 nm and **f** 400 nm, respectively. The directions of the incident light are marked on the left of the figures

A schematic diagram of the proposed bifunctional nanoparticles is shown in Fig. 1. The proposed nanoparticles consist of Eu-doped Gd_2_O_3_ nanospheres located in silica (SiO_2_) shell, as shown in the enlarged schematic diagram in Fig. 2a. For nanoparticles, the silica cladding has a lower refractive index (in visible-NIR wavelengths) and the Gd_2_O_3_ core has a higher refractive index. In order to obtain a localized light field, a resonant mode similar to the whispering gallery mode will be obtained, usually by adjusting its cross-sectional size. To this end, with the help of finite difference method (FDM) and finite difference time domain (FDTD) method, the effective mode field range and effective refractive index are examined as functions of nanocore and cladding radii, including *R* and *r*. The proposed nanoparticles are designed to operate at near-infrared wavelengths of ${\lambda }_{0}$ ≈ 800 nm. It is mentioned in some previously reported work that the thickness of the silica cladding is tens to one hundred nanometers. The size of the optimally functioning nanoparticle is basically determined by the effective mode field range and effective refractive index. Furthermore, by FDTD, the optical field localization at the working wavelength is determined. Considering that the size of the nanoparticles is much smaller than the laser spot cross section of the working wavelength, a plane wave is applied to the nanoparticles as the excitation light source.

### Synthesis of Gd_2_O_3_:Eu^3+^@mSiO_2_ nanoparticles

A urea-based homogeneous precipitation process was used to prepare Eu ion-doped Gd_2_O_3_ spheres, in which dosages of Eu ion doping were set to be 1, 1.5, 2, 2.5, 3, 3.25 wt%. A total of 0.75 mmol of Gd(NO_3_)_3_·xH_2_O and Eu(NO_3_)_3_·6H_2_O in a molar ratio of 98.5:1.5 (1 wt%), 97.7:2.3 (1.5 wt%), 97:3 (2 wt%), 96.2:3.8 (2.5 wt%), 95.4:4.6 (3 wt%), 95:5 (3.25 wt%) was weighed, then 1.5 g of urea and 50 ml of deionized water were added and the mixture was stirred for 2 h. After heating in a water bath at 90 °C for 2 h, the precursor powder was obtained by centrifugation and drying at 80 °C. After sonicating 0.1 g of solid powder with 2 ml of ethanol for 30 min and centrifuging, 40 ml of ethanol, 10 ml of deionized water and 0.5 ml of concentrated ammonia were added and the mixture was stirred for 30 min. Next, 16 µL of tetraethyl orthosilicate (TEOS) was dropped into the solution and stirred for 6 h. After centrifugation, the product was washed three times with water and ethanol; then, the product was collected. The product was dispersed in a solution containing 0.15 g of cetyltrimethylammonium bromide (CTAB), 40 ml of deionized water, and 30 ml of absolute ethanol with 0.6 ml of concentrated ammonia added and stirred for 30 min. Later, 210 µL of TEOS was dropped into the solution and stirred for 6 h. After stirring, the resulting products were collected and separated by centrifugation. They were then subjected to multiple washes with ethanol and water before being dried in air at 80 °C for 24 h. Subsequently, the dried samples were calcined at 550 °C for 6 h. The resulting product, with CTAB completely removed, was designated as Gd_2_O_3_:Eu^3+^@mSiO_2_ [44]. Finally, mark the product as Gd_2_O_3_:Eu^3+^@mSiO_2_. Additionally, all the chemical agents above used in this experiment were of analytical grade without further purification.

### Characterization

A Hitachi S4800 SEM operating at 3 kV was utilized for morphology and composition analysis. The particle size of the nanoparticles was determined using MATLAB, employing image processing techniques to generate a histogram of the particle size distribution. X-ray diffraction (XRD) patterns were acquired on a Bruker D8 Focus diffractometer with Cu-Kα radiation (*λ* = 0.15405 nm). To examine the composite's morphology further, the pore size of mSiO_2_ was measured using an accelerated surface area and porosimetry system (Micromeritics ASAP2460). For investigating the linear absorption region, an absorption spectrum of the nanoparticle samples was obtained using a UV/VIS/NIR spectrophotometer (UV-8000S, Shanghai Yuanxi instrument, Shanghai, China) over the range of 190–1100 nm, with a resolution of 1 nm. Magnetization measurements were conducted at room temperature using a VersaLab system (Quantum Design). Additionally, an electron paramagnetic resonance device (A300-10/12, Bruker, Germany) was employed to investigate the magnetic moment resonance of the samples.

### One- and two-photon excited luminescence and optical nonlinear

Room temperature photoluminescence (PL) spectroscopy of all nanoparticle samples was performed by a home-built optical spectrometer consisting of a 405 nm laser as the light source and a spectrometer (NOVA, Ideaoptics, Shanghai, China) as the detector. A Z-scan system was used to study the optical nonlinear properties of the nanocomposite samples. A home-made Z-scan system consists of an amplified femtosecond laser (Spectra-Physics Spitfire Ace with Mai Tai SP, repetition rate of 1 kHz, wavelength: 800 nm) with a pulse duration of 35 fs, repetition rate of 1 kHz, and wavelength of 800 nm. A lens with a focal length of 100 mm is used to focus the fs laser and collect the signal light. The sample is mounted on a motorized stage to pass through both lenses. The transmission through the sample is a function of the incident intensity and is detected by a photodetector located at the end of the system. To obtain stable two-photon excited luminescence, power-dependent PL spectroscopy measurements were performed by using the same amplified femtosecond laser system as the optical excitation source at 800 nm. The excitation power and polarization direction of the femtosecond laser are controlled by a tunable dielectric film attenuator and a pair of linear polarizers. The laser focused through a cylindrical mirror and focused on the surface of the nanoparticle sample. The nanoparticle powder is dispersed by ethanol and uniformly coated on a glass sheet (BK7). After drying, the sample is pressed and sealed with another piece of the same glass. An aspherical lens (35 mm focal length) was placed behind the sample to collect the two-photon pumped fluorescence excited by the fs laser. And a short-pass filter was passed to remove the signal from the fs laser. Finally, the obtained fluorescence signal was analyzed by a spectrometer.

### Experimental animal

All animal experiments were performed in accordance with China FDA guidelines. Protocols were reviewed and approved by the Scientific Research Ethics Committee of the Second Hospital of Shandong University. And four- to six-week-old male SD rats were purchased from Vital River Laboratory Animal Technology Co., Ltd.

### In vitro and in vivo MRI

The T1 relaxivity of Gd_2_O_3_:Eu^3+^@mSiO_2_ nanoparticles in phosphate-buffered saline (PBS) was assessed using a clinical Philips 3.0 T MRI scanner. Solutions of Gd_2_O_3_:Eu^3+^@mSiO_2_ nanoparticles with a doped dosage of 2.5 wt% at various concentrations (0.1, 0.15, 0.2, and 2.5 mM) were prepared by dissolving the nanoparticles in 1 mL of PBS. The MR signals of these solutions were acquired using a T1-weighted spin echo (SE) sequence with a TE of 15 ms and TR ranging from 20 to 1000 ms. The corresponding 1/T1 values were derived from the acquired T1-weighted MR images, and the relaxivity (r1) was calculated by plotting 1/T1 as a function of different Gd concentrations.

Healthy male SD rats (8–10 weeks old) were selected for liver MR imaging using the MRI scanner. T1 mapping sequence was employed for scanning, with the following parameters: TR = 1.35 ms, TE = 2.9 ms, 10 slices, 5.0 mm thickness, and FOV = 150 × 150 mm. Scanning was performed both before and 60 min after the injection of Gd_2_O_3_:Eu^3+^@mSiO_2_ nanoparticles in PBS at a dose of 10 mg/mL and a volume of 1 mL per 0.5 kg rat. The injection was manually administered through the tail vein.

### Cell culture and MTT experiment

Cell viability was assessed using MTT assays, with each experimental group consisting of multiple wells (6 wells per group) for parallel experiments. Cells in each group were treated with complete medium containing various concentrations (0, 5, 10, 15, 20, 25 µM) of nanoparticles for 2, 12, and 24 h, while the control group received an equal volume of PBS solution. After the designated incubation period, 20 µl of MTT reagent (5 mg/ml) was added to each well of a 96-well plate and incubated for 4 h. The reaction was stopped by adding 100 µl of dimethyl sulfoxide (DMSO) and incubating at 37 °C in the dark for 10 min. Cell proliferation was determined by measuring the absorbance at 490 nm.

Mouse bone marrow-derived MSCs (Invitrogen, USA) were cultured in DMEM-F12 medium (HyClone, Logan, UT, USA) supplemented with 10% FBS (HyClone), 1% gentamicin (GIBCO-BRL Life Technologies, Gaithersburg, MD, USA), and 1 × Glutamax (Invitrogen). Human hepatoma cell lines SMMC.7721 were obtained from the Chinese Center for Type Culture Collection (CCTCC, Wuhan, China) and cultured in Dulbecco's modified Eagle's medium (DMEM, HyClone, Logan, UT, USA) supplemented with 10% fetal bovine serum (FBS, Gibco, Grand Island, NY). Both cell types were incubated in a humidified incubator at 37 °C with 5% CO_2_. Mouse bone marrow-derived mesenchymal stem cells (BM-MSCs) were purchased from Invitrogen.

### Hematoxylin and eosin (HE) staining

HE staining was performed following standard protocols. Briefly, tissue sections were deparaffinized and rehydrated, and then stained with hematoxylin solution (ZSGB-BIO, China) for 5 min. Subsequently, the sections were dipped in 1% acid ethanol (1% HCl in 75% ethanol) five times and rinsed with distilled water. Eosin solution (ZSGB-BIO, China) was applied to the sections for 3 min, followed by dehydration using graded alcohol and clearing with xylene. Finally, the mounted slides were examined and photographed using a LEICA DM3000 LED microscope (Leica DMshare (v3), Germany).

## Results and discussion

First, the basic morphology of a structure is constructed, covering the inner core of Gd_2_O_3_ and the outer cladding of SiO_2_. According to the fabrication process of the samples, the SiO_2_ cladding thickness of the nanoparticles was set to be 100 nm (*R* − *r* = 100 nm). The outer diameter R is set to scan in the range of 200–400 nm, and in this range, according to the FDM results, a stable localized light field pattern in the Gd_2_O_3_ core can be obtained, as shown in Fig. 2b and c. On the basis of the results obtained by FDM, the effective refractive index and effective mode field area are functions of wavelength shown in Fig. 2d. In order to localize the laser with the working wavelength of 800 nm in the Gd_2_O_3_ core as much as possible to obtain more efficient light conversion efficiency, both the effective refractive index and the effective area of the mode field should be fully considered. However, when the inner diameter is too large, the nanoparticles which carries efficient localized modes cannot only stay in the core area. Therefore, a particle with an outer diameter of 400 nm is considered as the optimal size for bifunctional work. Considering the actual application scenario, further FDTD simulations were implemented. In the FDTD simulations, nanoparticles with an outer diameter size of 300–450 nm were constructed. By placing a plane wave with a wavelength of 800 nm on one side of the nanoparticle, the localized light field distribution in the nanoparticle was obtained when the light beam passed through the nanoparticle. Figure 2e and f shows the nanoparticle with outer diameter *R* values of 300 nm and 400 nm, respectively (the inner core regions are marked with black dashed lines). Both simulations set the incident light as a plane wave, with the direction of propagation from left to right, and are marked on the left side of both figures. By comparing the two figures, it can be clearly seen that when the outer diameter is 400 nm, the intensity of the local light field in the Gd_2_O_3_ core is stronger and the range is significantly larger. This result is similar to the previous FDM simulation result.

**Fig. 3:**
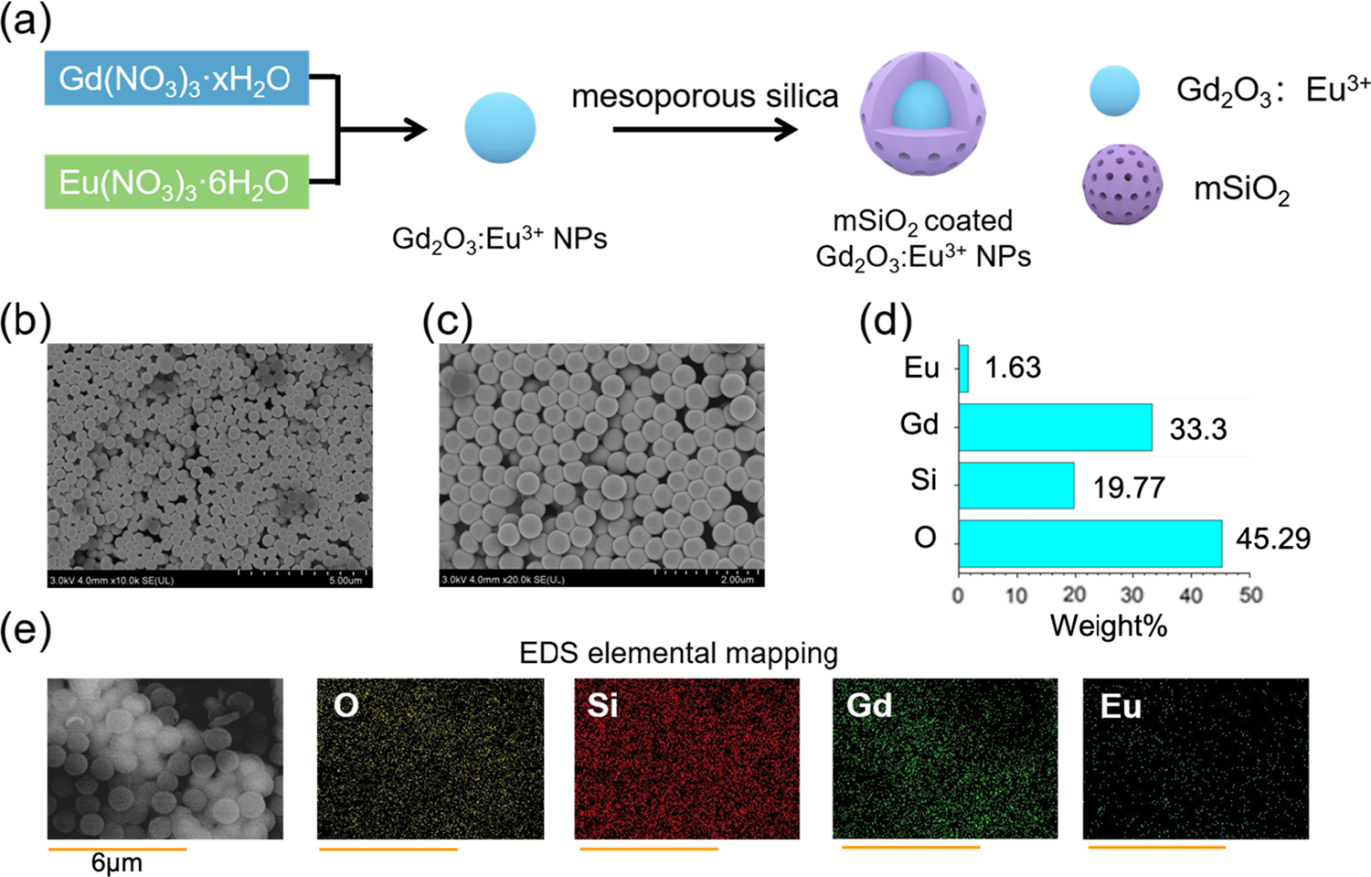
Characterization of Gd_2_O_3_:Eu^3+^@mSiO_2_ nanoparticle. **a** Scheme representing the synthesis of Gd_2_O_3_:Eu^3+^@mSiO_2_ nanoparticle. **b** SEM images of Gd_2_O_3_:Eu^3+^@mSiO2 nanoparticle with Eu ion dose of 3.25 wt%. **c** Smaller scale SEM images of the nanoparticle. **d** EDS weight percentage distribution of all elements in nanoparticle. **e** EDS element mapping of nanoparticle showing the presence and distribution of all elements

**Fig. 4:**
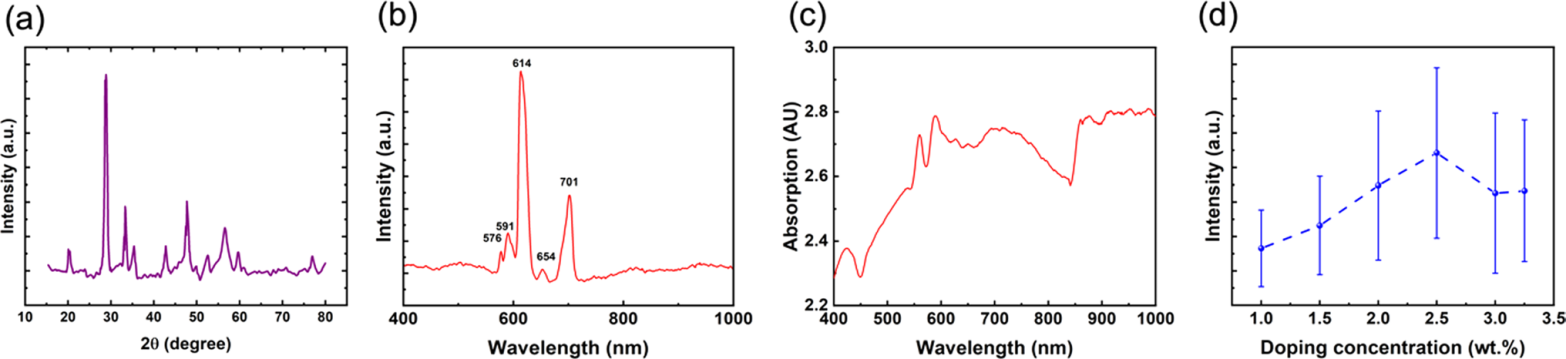
**a** Wide-angle XRD patterns of the Gd_2_O_3_:Eu^3+^@mSiO_2_ nanoparticles. **b** The emission spectrum of Gd_2_O_3_:Eu^3+^@mSiO_2_ nanoparticles under the excitation light of 405 nm. **c** The absorption spectrum of nanoparticles. **d** Intensities of 615-nm peaks emission at different concentrations of Eu^3+^ doping

The fabricating procedure of the Gd_2_O_3_:Eu^3+^@mSiO_2_ nanoparticle samples is shown in Fig. 3a. From the SEM images of the nanoparticle sample synthesized with Eu ion dose of 3.25 wt%, the samples consist of nanospheres with an average particle size of about 400 nm and their surfaces are smooth (Fig. 3b, c). The chemical properties of nanoparticle sample show the presence of Gd and Eu elements, and the distribution of elements is uniform. As can be seen in the high-angle annular dark-field scanning transmission electron microscopy (STEM) energy-dispersive spectroscopy (EDS), elemental distribution is about 33% for Gd and about 1.6% for Eu (Fig. 3d). Figure 3e shows elemental mapping, which is almost similar to EDS weight percentage distribution of Gd and Eu metal in the sample. Figure 4a illustrates the wide-angle XRD patterns of Gd_2_O_3_:Eu^3+^@mSiO_2_ and the standard data for Gd_2_O_3_ (JCPDS No. 86-2477). Upon thermal treatment at 550 °C for 8 h, the Gd_2_O_3_ particles within the silica matrix remain well-preserved, resulting in the formation of a cubic phase Gd_2_O_3_ [space group: Ia$\overline{3}$(206)] with a lattice constant of *a* = 1.08 nm.

The optical properties of nanoparticles were investigated by infrared–visible spectroscopy and fluorescence spectroscopy. As can be seen from Fig. 4b, the Gd_2_O_3_:Eu^3+^ nanoparticles were excited by visible light at *λ* = 405 nm, and the emission spectra were obtained. Between 400 and 1000 nm, five sets of emission bands corresponding to the transition of Eu^3+^ ion ^5^D_0_ → ^7^F_i_ (*i* = 0, 1, 2, 3 and 4) were obtained. The characteristic red light with the main emission peaking at 614 nm corresponds to the ^5^D_0_ → ^7^F_2_ transition of the Eu^3+^ ion. It belongs to the forced electric dipole transition of Eu^3+^ ions and indicates that Eu^3+^ is located in the lattice site with low symmetry and no inversion center in Gd_2_O_3_ matrix. Besides, there are other weak emission peaks of Eu^3+^ ions corresponding to transitions between energy levels, e.g., ^5^D_0_ → ^7^F_0_ (576 nm), ^5^D_0_ → ^7^F_1_ (591 nm), ^5^D_0_ → ^7^F_3_ (654 nm), ^5^D_0_ → ^7^F_4_ (701 nm) [45]. Figure 4c shows the absorption spectrum of Gd_2_O_3_:Eu^3+^@mSiO_2_ nanoparticles. Absorption spectra reflect the probability of transition of electrons from the ground vibrational level of the ground state to a specific vibrational level of the excited state after irradiation with light of a specific wavelength. The excitation energy is comparable to the absorption energy and infers the same information because the fluorescent molecule will reach the excited state at absorption. The absorption spectrum contains a broadband. To obtain the optimized doping concentration, the 615 nm excitation peaks of samples with different Eu^3+^ ion doping concentrations were tested at 405 nm excitation. Five different regions of the sample were excited for each concentration, ranging from 1 to 3.25%. Notably, 3.25 wt% was considered as the critical concentration for Eu^3+^ ion fluorescence quenching. It can be seen from Fig. 4d that the fluorescence intensity is stronger in range from 2.5 to 3.25%. Therefore, in the following pulsed laser excitation up-conversion experiments, samples with a concentration of 2.5% were selected.

**Fig. 5:**
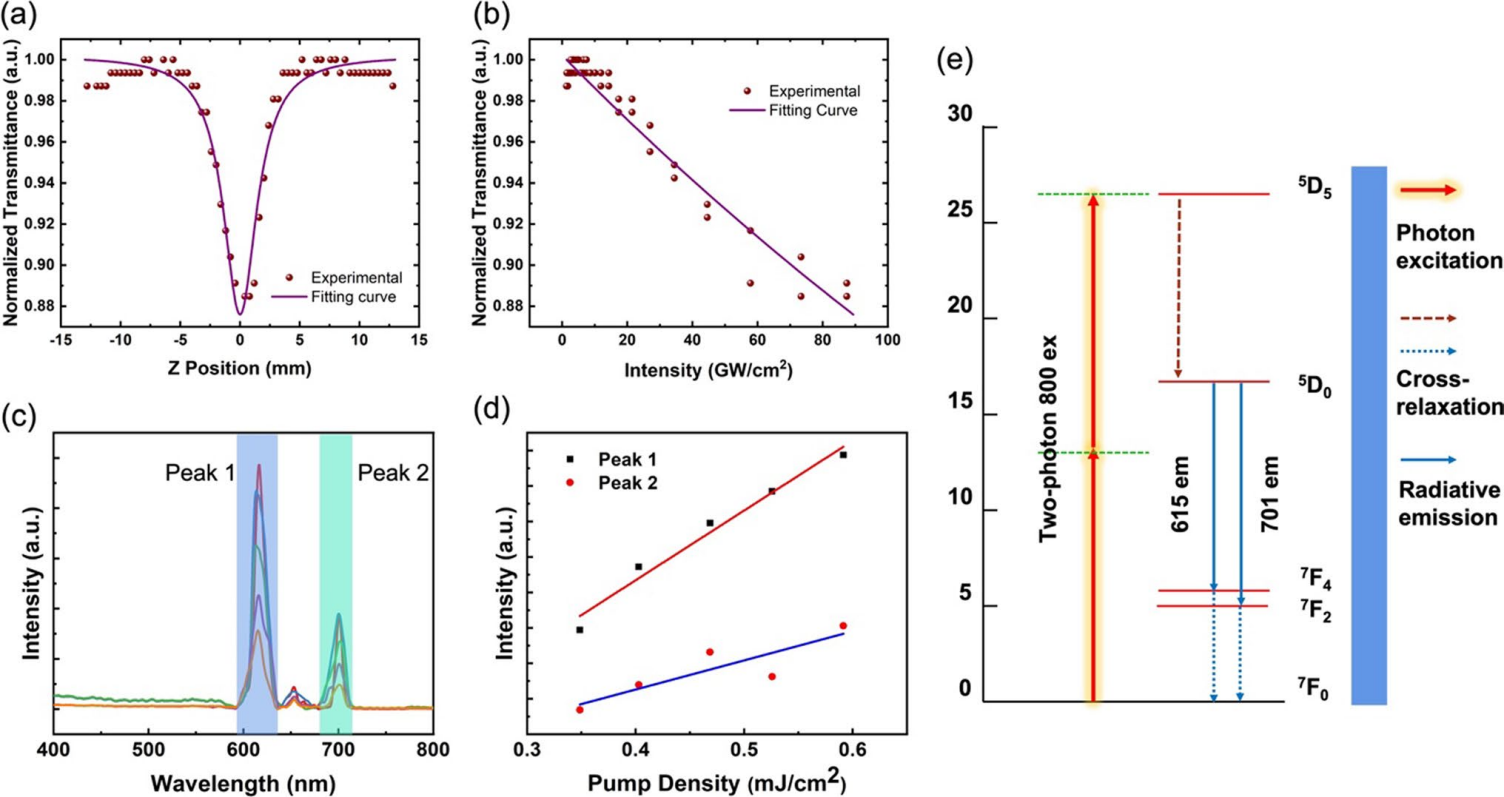
**a** The nonlinear optical properties of the sample measured by an open-aperture Z-scan system, with incident laser energy of 40 nJ. **b** Normalized transmission as a function of incident laser intensity. The scatters are experimental data and solid lines are the fitting curves. **c** Up-conversion of Gd_2_O_3_:Eu^3+^@mSiO_2_ nanoparticle under femtosecond pulsed laser excited with wavelength of 800 nm excitation. The pumped densities are from 0.35 to 0.59 mJ/cm^2^, and two mainly emitted peaks at 615 nm and 702 nm are labeled; **d** integrated intensities of 615 nm and 702 nm emitted peaks and their linear fitting. **e** Simplified energy-level diagram depicting the energy-transfer process of two-photon excited fluorescence of 800 nm pumping

Figure 5a shows a typical Z-scan curve of a nanoparticle sample that exhibits a distinct nonlinear response under excitation by an 800 nm fs laser at an excitation pulse energy of 40 nJ. These points are raw experimental data. The data show a symmetrical distribution of fluctuations to the central position (*z* = 0). The transmitted light intensity tends to decrease abruptly as the z position moves from the far to the central position. This fluctuation indicates a possible nonlinear response of the second-order nonlinear mechanism typical of two-photon absorption. Therefore, we used a formula to fit the data of the nonlinear response induced by the two-photon absorption mechanism. The formula is as the following [46, 47],

$$\frac{{{\text{d}}I}}{{{\text{d}}z}} = - \left( {\alpha_{0} + \beta I} \right) \cdot I$$

where *z* is the laser propagation distance in the sample, $\alpha_{0}$ is the linear absorption coefficient, *I* is the intensity and $\beta$ is the nonlinear absorption coefficient. The solid lines were fitting curves. After formula fitting, the value of $\beta$ was calculated to be 1.181 × 10^−2^ cm/GW, which indicated nonlinear response including two-photon absorption. Figure 5b depicts the transmittance as a function of the excitation light intensity by considering of the beam waist of the Gaussian beam.

Up-conversion luminescence is a widely recognized phenomenon that involves the conversion of near-infrared radiation (NIR) into visible light. This process, known as anti-Stokes luminescence, enables the conversion of two or more low-energy photons into a higher energy photon. In this study, we employed an amplified 800 nm femtosecond pulsed laser to excite up-conversion luminescence. The emitted spectra were then detected using a spectrometer within the 400–800 nm range as depicted in Fig. 5c. The spectra of nanoparticle samples indicated three emitted peaks at 615, 650 and 701 nm which correspond to the energy level transitions of Eu^3+^, and the peak positions were almost the same. The performances of 615 nm (Peak 1) and 701 nm (Peak 2) peaks are relatively stable under femtosecond laser pulse excitations. Both integrated intensities are exhibited in Fig. 5d. The integrated intensities of both peaks were linearly fitted and marked in the figure by the red and blue lines, respectively. As shown in the figure, peak 1 exhibits higher stability under femtosecond laser pulse excitation. The slopes of the linear fittings of the two peaks are slightly different, which might be due to the competitive luminescence mechanism between the energy levels of Eu ions under pulsed laser excitation in our speculation. The generation of peak 1 and 2 under femtosecond laser pulse excitation corresponds to a complex leap process between multiple energy levels, which can be postulated after absorption spectra and continuous photoexcitation spectra, as shown in Fig. 5e. Furthermore, it can be speculated that the ^5^D_0_ → ^7^F_2_ luminescence peak corresponding to the Eu ion-doped nanoparticles at 615 nm has the potential to be used for up-conversion bioimaging.

**Fig. 6:**
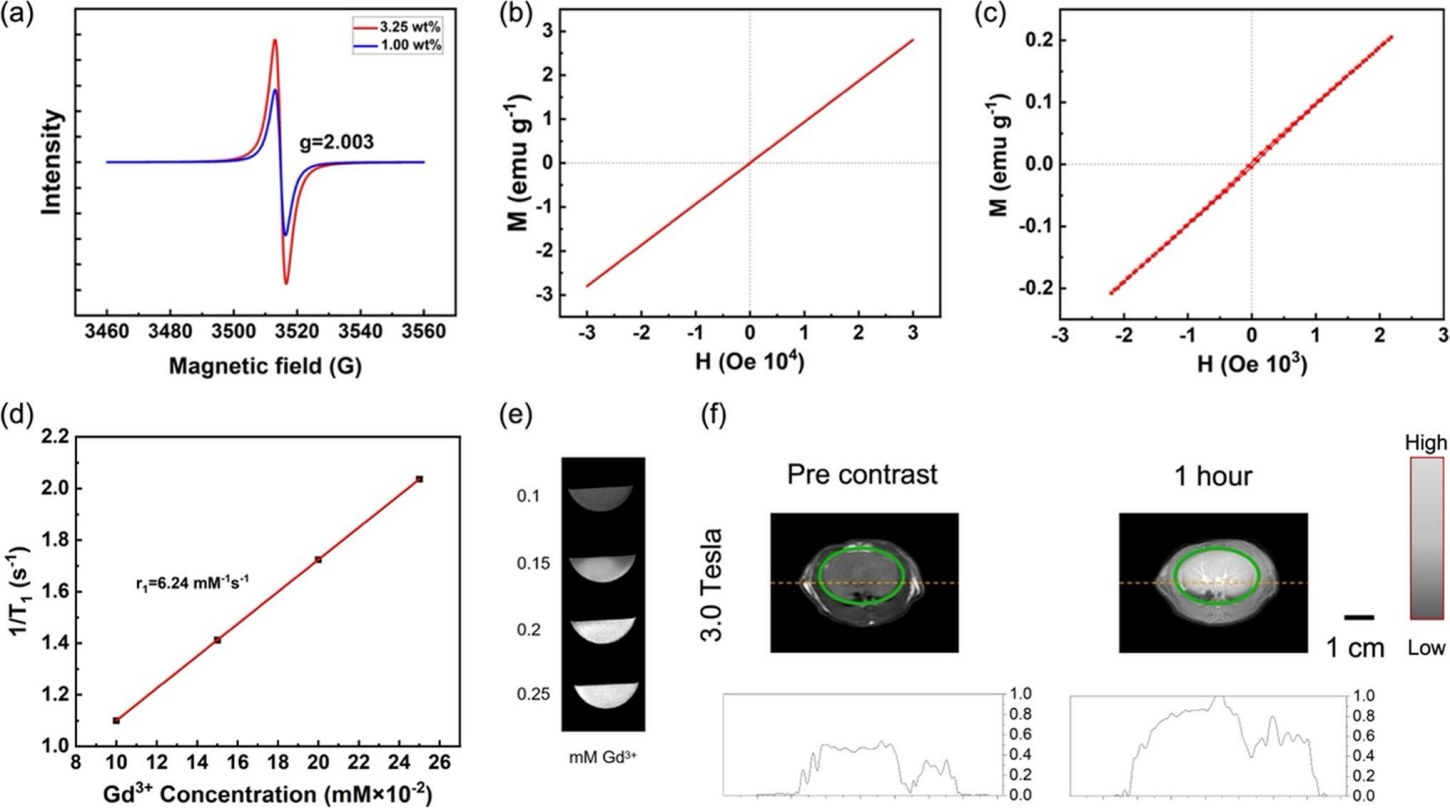
Measured magnetic properties and MR images. **a** Electron paramagnetic resonance testing of Eu ion doping concentrations of 1.0 wt% and 3.23 wt%. **b** Magnetization versus magnetic field (M–H) plots for the nanoparticles. **c** M–H map of nanoparticles at low magnetic field. **d** T1 MR relaxivity of Gd_2_O_3_:Eu^3+^@mSiO_2_ nanoparticles under 3.0 Tesla field MRI. **e** The MR images of different concentrations of the nanoparticle in vitro. **f** The rat before and 1 h after injected was imaged with in vivo MRI to detect liver tissue. The grayscale intensity curves in the figure below show the sections marked by the red dotted lines in the figure

The EPR properties of nanoparticles contains 1 wt% and 3.23 wt% Eu^3+^, respectively (Fig. 6a). It is evident from the figure that for these two samples with different doping concentrations, the ESR signal is a spectral line with fairly high symmetry. By fitting the Lorentzian formula, the Lande factor *g* value is obtained. The *g* factor of both samples is equal to 2.003, which is quite close to the free electron's Lande factor ge ~ 2.0023. The in-plane magnetic hysteresis (M–H) for the Gd_2_O_3_:Eu^3+^ nanoparticles sample is shown in Fig. 6b. The obtained room temperature M–H plot is a straight line with magnetization directly proportional to the applied magnetic field. Meanwhile, the sample cannot reach saturation magnetization even at 30 KOe magnetic field at room temperature. Figure 6c shows the M–H curve at low magnetic field (− 20 KOe ~ 20 KOe), and the coercive field and remanence close to zero (~ 10 Oe) can be obtained, which indicates that the sample behaves paramagnetic at room temperature. The paramagnetic properties of the samples are mainly derived from Gd_2_O_3_. And the paramagnetism of Gd^3+^ in Gd_2_O_3_ comes from the seven unpaired electrons in its atomic 4f layer. The 7 unpaired electrons are shielded by the outermost ^5^s_2_ and ^5^d_6_ electrons from the crystal field of the lattice. Therefore, the magnetic moments associated with Gd^3+^ are localized and non-interacting, resulting in paramagnetism [48, 49]. These properties lay the foundation for Gd_2_O_3_:Eu^3+^@mSiO_2_ nanoparticles to become magnetic resonance contrast agents [50, 51].

The T1 relaxation properties of Gd_2_O_3_:Eu^3+^@mSiO_2_ nanoparticles with different concentrations were evaluated on a clinical MR scanner at a magnetic field of 3.0 T to test their in vitro imaging effects. As shown in Fig. 6d, the signal intensity of the nanoparticles increases with the increase in Gd concentration. The relaxivity is calculated as the slope of the curve 1/T1 with respect to the gadolinium concentration. A linear fit of the T1 values measured at different solution concentrations yields a slope *r*1 value of 6.24 mM^−1^ s^−1^ under 3.0 T magnetic field. In comparison to commercially available Gd-based contrast agents, the *r*1 value obtained in our study appears to be higher than the reported values for Dotarem (5.6 mM^−1^ s^−1^), Magnevist (3.4–4.1 mM^−1^ s^−1^), and comparable to Omniscan (4.1 mM^−1^ s^−1^) [52–54]. This suggests that our Gd-based nanoparticles exhibit a relatively higher relaxivity, indicating their potential for enhanced contrast and improved efficiency in magnetic resonance imaging applications.

Figure 6e presents the imaging effects of different concentrations of converted Gd that the nanoparticle suspended in PBS by a clinical MR scanner in a test tube. To examine the potential of Gd_2_O_3_:Eu^3+^ nanoparticles in MRI, a clinical 3 T MRI scanner was used to detect MRI T1-mapping signals of nanoparticles in living rats to assess their suitability as MRI contrast agents. Liver tissue of healthy rats was used as the target to observe the MR imaging effect in this study. The rat liver tissue was gray before injection, as demonstrated in Fig. 6f. In particular, there was no difference in signal intensity between liver vessels and other liver tissues. After 1 h post-injection, the T1-mapping signal in the liver was significantly enhanced. While the rats were anesthetized, the signal did not diminish significantly in more than two hours. Through programming grayscale analysis of images, the MRI T1 signal of liver tissue was enhanced by approximately 30% compared to pre-injection. Differences in the MRI signal of the major vessels in the liver made them easy to distinguish. In addition, the MRI of other tissues in the abdominal cavity also obtained different degrees of enhancement. The T1 relaxation properties of Gd_2_O_3_:Eu^3+^@mSiO_2_ nanoparticles were evaluated under a magnetic field of 3.0 T and the nanoparticles were sufficient to generate satisfactory MRI signals.

**Fig. 7:**
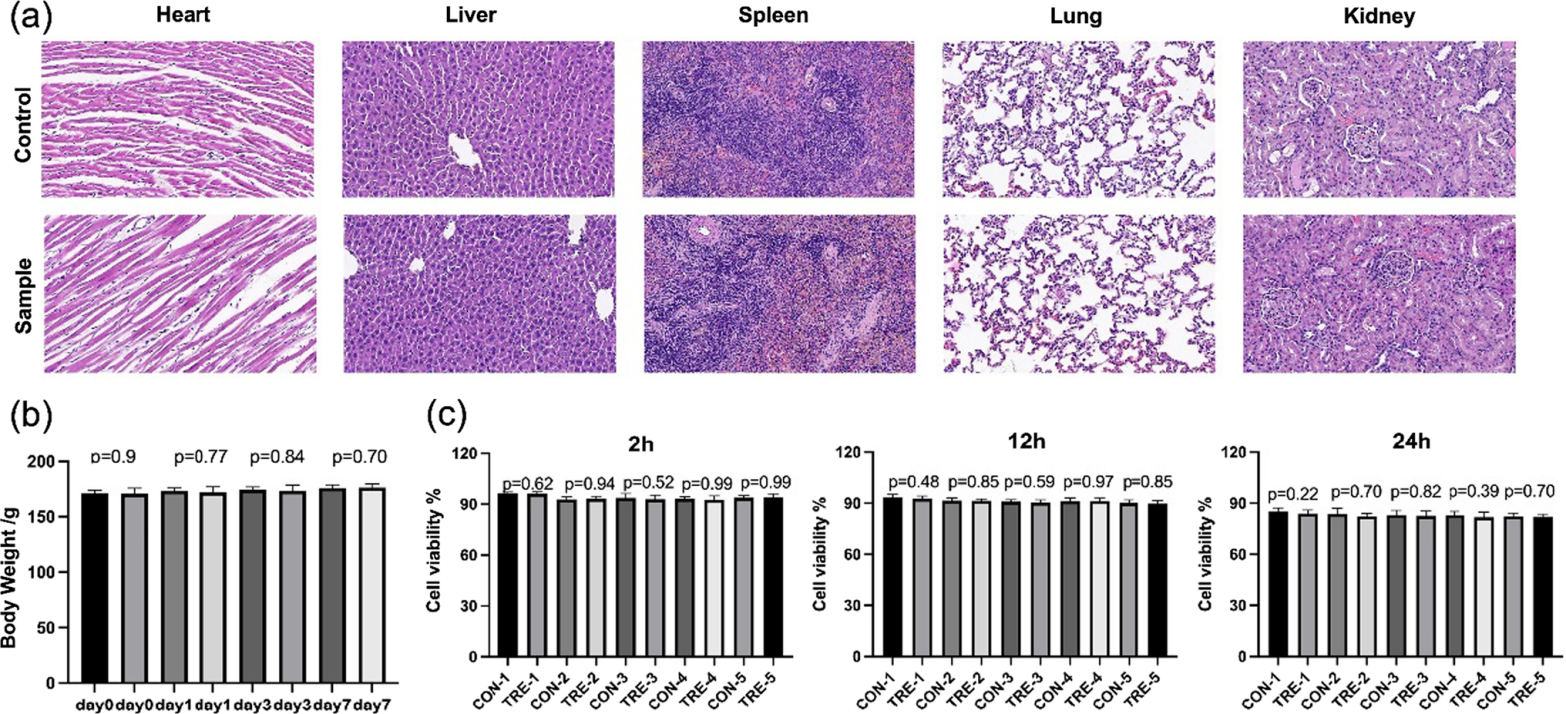
**a** H&E staining of major organs post-treatment. **b** Relative cell viabilities of HUVEC cells after various treatments at different concentrations (0, 5, 10, 15, 20 and 25 µM). The statistical analyses were conducted via a student’s *t*-test

**Fig. 8:**
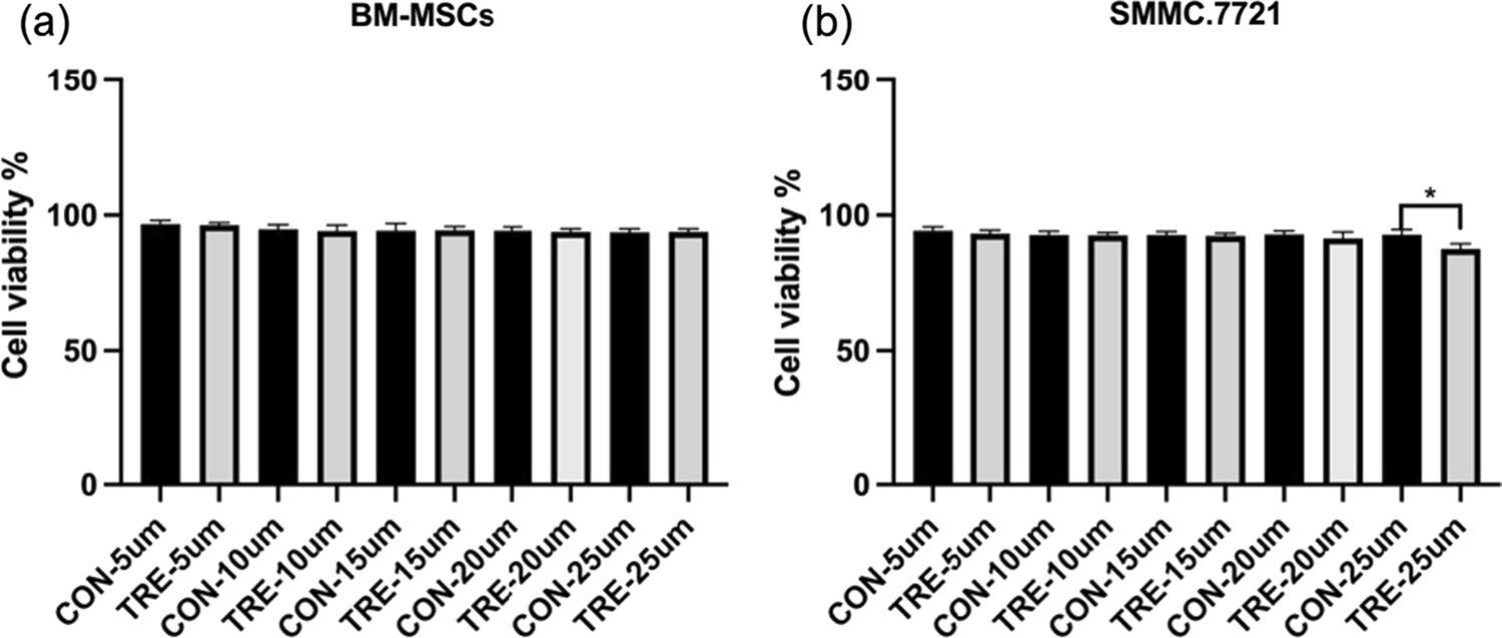
MTT assay of **a** BM-MSCs and **b** SMMC.7721 cells treatments for 24 h

To test the safety of nanoparticles in vivo and in vitro, we conducted cell and in vivo experiments. First, healthy SD rats were selected for intravenous injection of high dose (100 mg/kg) nanoparticle solution and the same amount of normal saline. After the injection, they were monitored for a week. During this period, no obvious change in the behavior of the animals in the experimental group was found, neither was obvious abnormalities in coat color and vitality. Hematoxylin and eosin (H&E) staining were used to make complete histological evaluation of the tissues on the organs (heart, liver, spleen, lung, kidney). H&E assessment did not reveal any significant changes in these tissues, indicating the absence of acute (7 day) toxicity following nanoparticle treatment (Fig. 7a). And there was no significant difference in body weight trends between the two groups (Fig. 7b). At the same time, we used nanoparticle solution to culture human umbilical vein endothelial cells (HUVECs) (Fig. 7c). Compared with the control group, there was no significant difference in cell activity at different concentrations and time gradients, indicating that the nanoparticle material had considerable safety. We evaluated the effects of NPs on the viability of BM-MSCs by co-culturing them for 24 h (Fig. 8a). Our findings showed that there was no significant decrease in MSCs' activity, indicating that NPs did not affect MSCs' viability. Subsequently, we investigated the impact of NPs on tumor cells. Co-culturing NPs with SMMC.7721 cells for 24 h revealed an interesting result: at a concentration of 25 µm, the survival rate of the NPs group was significantly reduced compared to the control group (Fig. 8b). This may be attributed to the higher uptake rate of NPs by cancer cells. However, a single cell toxicity study is insufficient to fully elucidate the potential anti-tumor capabilities of NPs. Therefore, we plan to design more comprehensive experiments to further investigate this characteristic in-depth.

## Conclusion

In conclusion, we designed and successfully fabricated a mesoporous SiO_2_ coated paramagnetic Gd_2_O_3_ nanoparticles doped with different concentrations of Eu^3+^. The optical localization mechanism of the nanoparticles subwavelength structure and optical nonlinearity of nanoparticles were studied. During this process, we used several methods like light field distribution, mode analysis and Z-scan technique. Based on research, we discovered the nanoparticles sample exhibits a nonlinear effect of two-photon absorption with a nonlinear absorption coefficient of 1.181 × 10^−2^ cm/GW. Two stable luminescence peaks of visible red light at wavelengths of 615 nm and 701 nm, respectively, appeared under the excitation of the 800 nm near-infrared femtosecond pulsed laser. EPR and M–H show that the nanoparticle is paramagnetic. In vivo MRI shows that the nanoparticles can significantly enhance the signal intensity in liver tissue. All of the above suggests that this sample is a promising contrast agent for near-infrared fluorescence imaging and MRI and it has great potential in diagnosis of various imaging techniques.

## Data Availability

The datasets used or analyzed during the current study are available from the corresponding author on reasonable request.

## Code availability

Source code is available upon request from the corresponding author.
